# Understanding the Long Haulers of COVID-19: Mixed Methods Analysis of YouTube Content

**DOI:** 10.2196/54501

**Published:** 2024-06-03

**Authors:** Alexis Jordan, Albert Park

**Affiliations:** 1 Department of Software and Information Systems UNC Charlotte Charlotte, NC United States

**Keywords:** long haulers, post–COVID-19 condition, COVID-19, YouTube, topic modeling, natural language processing

## Abstract

**Background:**

The COVID-19 pandemic had a devastating global impact. In the United States, there were >98 million COVID-19 cases and >1 million resulting deaths. One consequence of COVID-19 infection has been post–COVID-19 condition (PCC). People with this syndrome, colloquially called *long haulers*, experience symptoms that impact their quality of life. The root cause of PCC and effective treatments remains unknown. Many long haulers have turned to social media for support and guidance.

**Objective:**

In this study, we sought to gain a better understanding of the long hauler experience by investigating what has been discussed and how information about long haulers is perceived on social media. We specifically investigated the following: (1) the range of symptoms that are discussed, (2) the ways in which information about long haulers is perceived, (3) informational and emotional support that is available to long haulers, and (4) discourse between viewers and creators. We selected YouTube as our data source due to its popularity and wide range of audience.

**Methods:**

We systematically gathered data from 3 different types of content creators: medical sources, news sources, and long haulers. To computationally understand the video content and viewers’ reactions, we used Biterm, a topic modeling algorithm created specifically for short texts, to analyze snippets of video transcripts and all top-level comments from the comment section. To triangulate our findings about viewers’ reactions, we used the Valence Aware Dictionary and Sentiment Reasoner to conduct sentiment analysis on comments from each type of content creator. We grouped the comments into positive and negative categories and generated topics for these groups using Biterm. We then manually grouped resulting topics into broader themes for the purpose of analysis.

**Results:**

We organized the resulting topics into 28 themes across all sources. Examples of medical source transcript themes were *Explanations in layman’s terms* and *Biological explanations*. Examples of news source transcript themes were *Negative experiences* and *handling the long haul*. The 2 long hauler transcript themes were *Taking treatments into own hands* and *Changes to daily life*. News sources received a greater share of negative comments. A few themes of these negative comments included *Misinformation and disinformation* and *Issues with the health care system.* Similarly, negative long hauler comments were organized into several themes, including *Disillusionment with the health care system* and *Requiring more visibility.* In contrast, positive medical source comments captured themes such as *Appreciation of helpful content* and *Exchange of helpful information.* In addition to this theme, one positive theme found in long hauler comments was *Community building.*

**Conclusions:**

The results of this study could help public health agencies, policy makers, organizations, and health researchers understand symptomatology and experiences related to PCC. They could also help these agencies develop their communication strategy concerning PCC.

## Introduction

### Background

“It’s like a...like a viral tornado that goes in you and kind of just messes you up,” Sadi Nagamutu says in between labored breaths [1]. This is how the account of the battle with post–COVID-19 condition (PCC) of Sadi Nagamutu, a fitness instructor aged 44 years, began during a news interview [1]. In the comment section of the video, one user wrote the following:

I had to pause the video at 2:20. I broke down in tears because I feel like I’m not alone. I have the same thing.

At the time of recording, Sadi Nagamutu had been a patient with PCC for 8 months. By this time, she claims that it had completely disrupted her life. She notes that she went from being a trainer to not being able to lift grocery bags and walk at the same time [1]. It is clear from the comments left under the 60 minutes video that Sadi Nagamutu is not alone in experiencing a drastic change in her quality of life.

The COVID-19 pandemic has changed the lives of many, though one consequence of it has received less attention [2]. PCC has been identified as a syndrome affecting patients long after their initial COVID-19 infection has cleared. These patients are colloquially called *long haulers* [3]. High ratios of those who have been infected with COVID-19 have persisting symptoms that last months after the initial infection.

Studies have shown that PCC has real implications in people’s everyday lives. The World Health Organization Quality of Life Brief Version, a quality of life questionnaire, was administered among patients who had been hospitalized with COVID-19 [4]. The results showed that 30.2% of respondents had PCC, which affected nearly all domains of quality of life as outlined in the World Health Organization Quality of Life Brief Version criteria [4]. Moreover, there have been recent links between PCC and deteriorating mental health [5].

PCC has negative economic implications as well. Those with PCC are often not in condition to work and, thus, realize their full earning potential [2]. Approximately 44% of people with PCC are completely out of the workplace, whereas 51% have reduced hours at work [2]. This could result in >US $50 billion in lost income annually [2].

In addition, some patients do not receive insurance coverage for PCC-related testing and treatments [6]. This has led to significant debt for some patients [6]. In May 2023, the *Journal of the American Medical Association* estimated that average PCC-related medical costs could be approximately US $9000 a year [6]. There is also the issue of lost wages due to PCC, which further complicates the medical debt. Making a case for those with PCC by uncovering patient experiences could be useful for public health officials and medical insurance companies, who may need additional help in understanding how debilitating PCC can be.

Social media is a rich source of information regarding people’s experiences and attitudes [7,8] due to the pervasiveness of social media apps and the freedom with which people engage in discourse on various topics. Such pervasiveness contributes to the increased size of health-related data [7]. This has encouraged researchers to use computational models to analyze social media texts concerning COVID-19 [7,9-12]. One popular method of analysis is topic modeling. Topic modeling allows for the discovery of thematic relationships and patterns within a body of text using natural language models [10]. Latent Dirichlet allocation (LDA) is a probabilistic unsupervised classification method [13]. It has been widely used in studies using topic modeling on a large set of documents [13].

For example, Mutanga and Abayomi [10] used an LDA topic model to study COVID-19–related posts in South Africa and found that conversations revolved around *alcohol consumption*, *staying at home*, *vaccine conspiracy* *theories*, *police brutality*, *statistics training*, and *5G* [10]. In addition, other authors have explored public sentiment and discourse on COVID-19 vaccines on Reddit using an LDA model [9]. They found that posts covered the broader discussions of *vaccines*, *safety* *concerns*, *efficacy*, and *side effects*.

To date, there has been one other study that examined the experiences of long haulers on YouTube in the hopes of understanding web-based health communication [7]. However, Jacques et al [7] did not use any topic modeling methods. Instead, they manually coded the 100 most viewed PCC videos based on a predetermined list of themes.

In the following sections, we provide a review of long haulers and health discussions on YouTube. On the basis of this, we sought to understand what types of videos are available on YouTube regarding long haulers and how users respond to PCC-related content. Next, the procedure of data collection and analysis is provided. Results are then reported regarding salient themes for each type of content creator and positive and negative comments. Finally, we conclude with a discussion of the theoretical and practical implications of our findings.

### Related Work

#### Long Haulers

Studies have focused on long haulers in the hopes of understanding their symptoms and concerns [14-16]. By analyzing Reddit posts, Thompson et al [15] found that discussions revolved around *symptoms*, *diagnostic* *concerns*, *broad* *health* *concerns*, *chronicity*, *support*, *identity*, and *anxiety*. In the study by Basch et al [17], news articles and videos were selected from a news media platform (Google News). They were then analyzed to identify common symptoms that appeared in PCC-related content [17]. The authors found that 41% of news reports mentioned the *duration* of the symptoms, which tended to range between 1 month and over a year. *Tiredness* and *fatigue* were the most mentioned symptoms, occurring in 74% of the news content. Though insightful, these studies do not focus solely on the YouTube platform, wherein there can be interaction between the creators of long-form content and those consuming their media.

#### Health on YouTube

YouTube is a platform that motivates users to create, publish, and comment on posts [18]. It has been developed to handle long-form content. YouTube is unique in that creators of long-form content can not only share their videos but also engage with viewers within the comment section. A report from Statista estimates that, as of April 2022, YouTube has 247 million users in the United States [19]. There have been studies in which researchers analyzed YouTube comments and transcripts to understand public sentiment on health-related matters. These studies have used either manual [20,21] or natural language processing–based [22,23] approaches.

Noncomputational analyses of YouTube videos have involved manually coding videos into various groupings. One study on anorexia-related YouTube videos used the help of 3 physicians to categorize 140 videos against a predetermined list of classification criteria [20]. In addition, to understand discourse on YouTube videos that seeks to destigmatize mental health, McLellan et al [21] manually coded 100 randomly selected comments from 20 videos based on predetermined coding criteria.

In contrast, Aslam et al [22] used computational methods to understand the transcripts of 1000 COVID-19–related YouTube videos [16]. They used the Gensim LDA topic model to understand the transcripts. They found that salient topics involved *symptoms*, *precautions*, and *home* *remedies* [22]. In their study, Serrano et al [23] fine-tuned the Robustly Optimized Bidirectional Encoder Representations Approach base to label comments from factual and misinformative COVID-19–related videos. In addition, they extracted features from video titles and comments [23]. These features were used in a linear support vector machine to detect misinformative videos [23].

We collected YouTube transcripts and comments between August 3, 2020, and October 29, 2021, to investigate PCC symptomatology and other related complications. We chose to use computational methods, more specifically topic modeling, because they can capture a wider distribution than manual studies [10]. To the best of our knowledge, this is the first study to examine YouTube video transcripts and comments related to PCC experiences. Our research questions (RQs) for this study were two-fold: (1) What types of videos are available on YouTube regarding PCC? (RQ 1) (2) How do users respond to PCC content? (RQ 2).

## Methods

### Data Collection

YouTube is a free-to-use social media platform that has been adopted by individuals, organizations, and specialized professionals from various fields to share relevant and important information [18]. Because of this, we deemed YouTube to be a good source of data to capture videos uploaded by different types of content creators. This allowed for diversity in our data set.

We used Google’s application programming interface, googleapiclient.discovery, to capture video comments and metadata (eg, number of comment likes and responses). Data from the top 50 videos as a result of searching each of the following terms were collected: “Covid Long Haulers,” “COVID-19 Long Haulers,” “Long Covid,” “Long Haul Covid,” “PASC Covid,” “Post-Covid Symptoms,” and “Post-Covid Syndrome.”

The search terms were found by first inspecting COVID-19 long hauler–related news articles to find pertinent keywords. After this, Google Trends was inspected to see whether there were any additional terms or versions of terms that had already been identified. We used these terms to find and inspect an initial list of videos on YouTube. After completing this process, we were able to rule out the term “Longhauler” as many references were not related to COVID-19. The resulting videos were in the date range between August 3, 2020, and October 29, 2021. The videos were collected on November 1, 2021. After removal of duplicates and irrelevant videos, we collected 152 videos.

We used the Python package *YouTubeTranscriptAPI* (Python Software Foundation) to capture transcripts from the videos. It should be noted that the comments collected in our data-gathering process only reflect the top-level comments. In essence, this means that any replies to the original comments were not captured.

We then manually grouped the videos based on the video source as previously done in a similar study [24]. This is because the topic coverage of videos can vary widely depending on the source of the video. The resulting groups were news sources, medical sources, and long haulers. News source videos were those that were uploaded by news entities, including local, national, and international news stations. News source videos represented 51.3% (78/152) of the collected videos. Medical source videos were those that were posted by medical experts such as physicians, health insurance companies, and medical schools. We collected 49 such videos. The last 16.4% (25/152) of the videos belonged to the long hauler grouping, which represented first-person accounts from those who considered themselves to have PCC. From these videos, we collected 2845 comments in total: 1258 (44.22%) associated with medical source videos, 1078 (37.89%) from news source videos, and 509 (17.89%) from long hauler videos.

### Ethical Considerations

We only analyzed publicly available documents in this study and did not analyze identifiable private information or involve any direct or indirect interactions with individuals. Per (blank for review) policy (citation: 45 Code of Federal Regulations 46 Definitions), this study is exempt from institutional review board requirements because it does not meet the regulatory definitions of human participant research. However, we removed any user identifiable information (eg, usernames) and paraphrased or modified comments to preserve user pseudonymity while maintaining the content’s integrity in the manuscript.

### RQ 1 Methods: What Types of Videos Are Available on YouTube Regarding PCC?

To understand themes within the video content, we used Biterm to generate topics of video transcripts as well. Biterm topic model learns topics by modeling the generation of word co-occurrence patterns in whole documents to counter the sparse word co-occurrence pattern problem that occurs when evaluating at the document level [25]. Each group—medical sources, news sources, and long haulers—was processed individually to preserve our groupings. Biterm was created with shorter social media texts in mind given that they are usually much shorter than standard document sizes [25]. Because video transcripts were considerably longer and, thus, could contain multiple topics, chronological batches of 50 consecutive words were fed into each model as suggested by previous work on topic modeling [26]. It was important to divide the transcripts into shorter portions so that more specific topics would be generated. After preprocessing our data by lemmatizing words and removing stop words, we fed our data into the topic models. To preserve our groupings, we created 6 separate models: one positive and one negative model for each group (news sources, medical sources, and long haulers). When fine tuning the number of topics, we tested 4 numbers (3, 5, 10, and 15). For each number, we assessed the coherence scores and strength for words within the same topic co-occurring in the same documents [25]. Biterm adopted a coherence score proposed by Mimno et al [27]. In the study by Yan et al [25], the average coherence score for a Biterm model with 5 topics was between −52.3 and −52.5. A limitation of the coherence score is that it only accounts for the most frequent topic words. To compensate for this limitation, we complemented the evaluation with manual analysis in addition to considering coherence scores for selecting the most cohesive model. To elicit unknown, emerging themes grounded in the labeled topics, we further qualitatively analyzed comments or transcripts within each topic following an open coding procedure [28] similar to that in a previous study that analyzed social media content that included YouTube videos on COVID-19 [29]. Following the collaborative identification of a list of topic labels, the research team independently labeled each topic using up to 50 most salient terms and up to 30 samples of the most representative content followed by grouping the topics into themes. At each iteration, the research team resolved any discrepancies through discussion.

### RQ 2 Methods: How Do Users Respond to PCC Content?

We conducted sentiment analysis to understand public sentiment with regard to the delivered content. We used the Valence Aware Dictionary and Sentiment Reasoner (VADER) [30] to determine the sentiment of video comments. VADER is a rule-based model for sentiment analysis. It was created specifically for social media contexts as it can recognize slang and emojis. It produces positive, negative, neutral, and compound scores for each body of text by summing the valence scores of each word and normalizing them to be between −1 and 1. We chose VADER in lieu of other sentiment analysis tools such as AFINN, BING, or National Resource Council because VADER was specifically developed for analyzing social media texts.

We used the compound score as the overall sentiment score for the comment. Positive comments included all comments with a compound score of >0. Negative comments included all comments with a score of ≤0 following methodological guidance from a previous study [31]. This process was completed independently for each group (medical sources, news sources, and long haulers).

After we created positive and negative subgroups of the comments, we created topic models to understand the thematic make-up of positive and negative comments with regard to each group. We separated comments into positive and negative subgroupings before generating topics so that our resulting topic models would be more cohesive. Similar to the methods for RQ 1, we used Biterm to generate topics and manual review to label topics and group them according to themes. Because comments are relatively short in length and typically have 1 topic, we used the entire comment as a document.

## Results

### Overview

We organized the resulting topics into 28 themes across all sources. Medical source transcript themes were *Explanations in layman’s terms*, *Show housekeeping*, and *Biological explanations*. News source transcript themes were *Sharing patient experiences*, *Negative experiences*, *Experts weighing in*, and *Handling the long haul*. Long hauler transcript themes were *Taking treatments into own hands*, *Changes to daily life*, and *Choosing homeopathy over pharmaceuticals*. Positive news source comment themes were *Extending empathy*, *Expressing distrust through sarcasm*, and *Encouragement for better outcomes*. News source videos received the highest proportion of negative comments. Negative news source comment themes were *Reproduction of debunked and political theories*, *Misinformation and disinformation*, and *Issues with the health care system*. In contrast, medical source videos received the highest proportion of positive comments. Positive medical source comment themes were *Appreciation of helpful content*, *Hope and encouragement*, and *Exchange of helpful information*. Negative medical source comment themes were *Negative impacts of long haul*, *Requiring medical alternatives*, and *Lack of needs*. Positive long hauler comment themes were *Appreciation*, *Exchange of helpful information*, and *Community building*. Negative long hauler comment themes were *Exchange of additional information*, *Disillusionment with the health care system*, and *Requiring more visibility*.

### RQ 1 Results: What Types of Videos Are Available on YouTube Regarding PCC?

#### Overview

We collected the transcripts from 152 videos that were divided into 3 groups (news sources: n=78, 51.3%; medical sources: n=49, 32.2%; and long haulers: n=25, 16.4%). Transcripts were divided into subgroups of 50 consecutive words and fed into distinct Biterm topic models. The following sections show the breakdown of videos by source type.

#### Medical Source Video Transcripts

##### Overview

The medical source video transcripts were captions from videos created by an individual or organization in the medical sector. This included physicians, medical insurance companies, and medical schools.

##### Explanations in Layman’s Terms

The first theme, *Explanations in layman’s terms*, covered 3 topics: “Symptomatology,” “Symptom etiology,” and “Symptom management.” As implied by the theme title, the transcript snippets constituting each topic displayed scientific speech that was relatively easy for the public to understand. The first topic, *Symptomatology*, covered video transcripts in which the speaker explained the symptoms associated with COVID-19. Some medical source content creators dedicated entire videos to just a few symptoms or a particular health system, as was the case in a video from University of Alabama at Birmingham medicine dedicated to PCC and hair loss:

...when you go through something stressful and you have a telogen effluvium, most of your hairs can enter the resting phase at the same time.

The second topic, “Symptom etiology,” featured transcript snippets that offered explanations of how PCC symptoms might have originated. Finally, “Symptom management” featured transcript snippets wherein medical professionals offered potential treatments for symptoms.

##### Show Housekeeping

*Show housekeeping* was another prevalent theme in medical source video transcripts. Associated topics were “Introducing the show or guest,” “Validating guests’ credentials as a reliable source,” and “Encouraging the audience to keep in touch.” As the name suggests, these videos routinely introduced each of the medical experts on the show and expounded on their credentials. This could potentially be due to the idea that many information consumers can be critical of the source of their information. Expounding on the guest speakers’ credentials could help build credibility and trust between the video publisher and the audience. The next topic dealt with encouraging the audience to keep in touch. Some medical source content creators offered links to other social media platforms where they could continue the long hauler conversation with engagers.

##### Biological Explanations

In general, biological explanations comprised transcript snippets that displayed more advanced scientific language than that shown in the *Explanations in layman’s terms* theme. Biological explanations featured 2 distinct topics: “Immunophenotyping” and “Explaining the mechanics of immune responses.” Immunophenotyping is the process of identifying cells based on antigens or markers [32]. In one video, the speakers discussed using “proprietary spark dyes,” which can be used for immunophenotyping [32]. In addition, these videos were concerned with explaining the mechanics of immune responses. In this case, a biological perspective of disease etiology was offered, with less use of layperson terminology.

#### News Source Video Transcripts

##### Overview

The news source video transcripts were the captions from news media outlets. These outlets ranged from local to international audiences (Table 1).

**Table 1:** News source video transcript results.

Theme and topic label		Keywords	Sample transcripts
**Sharing patient experiences**			
	Symptoms	“Patients,” “symptoms,” “life,” “understand,” “hair,” “feel,” “medical,” “sick,” “protein,” “heartbeat,” “health,” and “doctors”	“Five months later, she is still short of breath. Doing therapy three times a week. It often feels like this body is not mine. That the things that i want to do i can’t do.”
	Treatments	“Need,” “better,” “understand,” “doctors,” “months,” “trying,” “research,” “care,” and “answers”	“[...] even though there’s not a magic pill yet, to cure a long COVID, at least we can try to aggressively manage the symptoms, connect them with other patients, other resources, and try to help in whatever way we can.”
**Negative experiences**			
	Not being believed by others and doctors	“Symptoms,” “covid,” “virus,” “physician,” “dr,” “feeling,” “need,” “progress,” and “says”	“[...] to those doctors that deny the existence of long covid that this thing of course it’s really look at the science.”
	Explaining the impact of “long Covid” on lives	“Started,” “need,” “progress,” “end,” “taken,” “coming,” “time,” “medical,” “virus,” “smell,” “health,” “watch,” and “feeling”	“Differently, less like the flu and more like a condition that can have lasting repercussions. The moment [...] the sick get to go home. But for many it’s not the end, it’s just the beginning of a long and perilous road to recovery.”
**Experts weighing in**			
	Etiology of the disease	“Effects,” “infection,” “different,” “virus,” “actually,” “research,” “seen,” “syndrome,” “persistent,” and “fatigue”	“Today chris hrapsky talked with an expert whose theory on this is gaining attention. Mast cells are the first responders of your immune system when an infection occurs in under a second these cells and stuff like histamine to other cells to say, hey, wake up, something’s wrong here. In some people these mass cells go haywire and overreact like central dispatch calling in the swat team for a coffee spill at starbucks and this is called mast cell activation syndrome.”
	Experts explaining “long Covid”	“Struggle,” “lingering,” “illness,” “health,” “syndrome,” “persistent,” “group,” “body,” “covid19,” “physical,” and “related”	“The other thing that makes it really challenging, is that symptoms are not necessarily always correlated or equal to organ dysfunction that we can measure [...].”
**Handling the long haul**			
	Managing symptoms	“Test,” “hair,” “brain,” “doctor,” “fatigue,” “pain,” “disease,” “talk,” “common,” “exercise,” “need,” “home,” and “levels”	“[...] they sent an occupational therapist to see what they could do in the house so our washroom has been retrofitted with a brand new high toilet because he had issues getting on and off the toilet [...].”
	Handling cardiac or chest problems specifically	“Oxygen,” “need,” “lung,” “blood,” “chest,” “pain,” “infection,” “shortness,” “ventilator,” “loss,” “pulmonary,” “complications,” “attack,” “disease,” and “breath”	“[...] what i suggest is that those of our patients who are having tachycardia it’s not a bad idea to get themselves screened by their physicians or cardiologists so that at least we are clear that a patient does not have baseline pulmonary embolism [...].”

##### Sharing Patient Experiences

“Symptoms” and “Treatments” are 2 topics that were part of the *Sharing patient experiences* theme. The *Symptoms*-related video transcripts dealt with interviewees sharing their daily symptoms to give perspective to audiences. Interviewees experienced a wide range of symptoms. These symptoms appeared to have a significant impact on daily life. One interviewee noted that she would fall due to elevated heart rate that worsened doing routine tasks such as “just walking from here to the kitchen.” Guests were also concerned with finding some type of treatment that could mitigate PCC symptoms. Patients seemed to have managed expectations regarding treatment but exhibited some level of hope:

...there’s not a magic pill yet, to cure long Covid...at least we can try to aggressively manage the symptoms.

##### Negative Experiences

The *Negative experiences* theme featured 2 related topics. The first topic was “Not being believed by others and doctors*.*” This was a particularly common topic throughout the text. Interviewees shared their experiences of being ignored or not believed. These long haulers sought and could not find affirmation:

...no one really understands me.

The next topic dealt with explaining the impact of PCC on lives. Long haulers and news reporters introduced PCC in general terms as well as the people that it had impacted. One long hauler explained the following:

...nearly seven months later and I’m still unwell and I am still a broken woman.

##### Experts Weighing In

The *Experts weighing in* theme had 2 topics: “Etiology of the disease*”* and “Experts explaining long Covid*.*” Similar to medical source videos, experts took 2 approaches when speaking about PCC. The first approach, as evidenced in the *Etiology of the disease* topic, explained things from a strictly biological perspective:

Mast cells are the first responders of your immune system when an infection occurs.

In contrast, in *Experts explaining long Covid*, more commonly used colloquial language was used to explain PCC:

...different studies use different thresholds, which makes it really challenging to compare apples to apples.

##### Handling the Long Haul

The last theme had 2 topics as well: “Managing symptoms” and “Handling cardiac or chest problems specifically.” *Managing symptoms* dealt mainly with long haulers finding their own ways to manage their illness. In addition, cardiac and chest problems were often discussed. They are common symptoms that were addressed by experts and patients alike. Experts offered symptom management advice:

...and it will take three to six months for this myocarditis to settle.

#### Long Hauler Video Transcripts

##### Overview

The long hauler video transcripts were captions from individual content creators that talked directly about their own personal experiences with PCC (Table 2).

**Table 2:** Long hauler video transcript results.

Theme and topic label		Keywords	Selected sample transcripts
**Taking treatment into own hands**			
	Alternate remedies	“New,” “health,” “try,” “better,” “care,” “fungus,” “changing,” “trusted,” and “broken”	“Covid was my wake-up call to fix my gut and ultimately fix my health I was declining I was already declining before covid I was getting weak [...].”
	Dealing with uncertainty	“Changing,” “declining,” “shitty,” “new,” “life,” and “work”	“[...] this is my story right like this is this is what I have to live with for an indefinite period of time so my very good family friend she runs her own practice she’s an MD and she said you know like nobody should want to get Covid because nobody knows the lasting effects of Covid.”
	Not being listened to by physicians	“Biases,” “trusted,” “chore,” “dr,” “doctors,” “feel,” “medicine,” and “care”	“[...] especially female patients and patients of color the benefit of the doubt [...] there is so much research on patients reporting doctors not believing them or not treating them with the same level of compassion [...] I didn’t think it would happen to me [...].”
**Changes to daily life**			
	Insomnia	“Helped,” “started,” “pills,” “prevent,” “restless,” “waking,” and “blockers”	“I am allowed to take a maximum amount of the sleeping aids and they don’t work I just get a calming feeling along with my multitude of symptoms I think along with the drenching sweats and the fevers that just won’t stop because my husband has to cover me in ice sometimes because even with medication the fever doesn’t stop climbing.”
	How symptoms interrupt activities	“Day,” “symptoms,” “time,” “feel,” “bad,” “need,” “breath,” “overgrowth,” “taste,” “chronic,” “fever,” “life,” “nuts,” and “sacrifice”	“I had to stop eating eggs I recognize that eggs weren’t agreeing with me anymore and [...] I was eating three eggs every day like that was you know that was a breakfast staple for me [...].”
	How symptoms present themselves	“Experience,” “fever,” “health,” “day,” “highly,” “discovering,” “seizures,” “entry,” and “permanent”	“So like whenever i would get near like the oven or the stove or like the air fryer or take a shower or try to exercise like whenever my internal body temperature would rise my face would go bright red it would get swollen id’ get like weird patches it was super strange [...].”
**Choosing homeopathy over pharmaceuticals**			
	Use of CBD^a^ and THC^b^	“Gummies,” “high,” “work,” “try,” “started,” “need,” and “help”	“The cbd and thc gummies that I take to sleep at night [...] I just try to keep things as natural.”
	Turning down over-the-counter medicine	“Deficiency,” “vitamin,” “blood,” “different,” “taking,” and “bad”	“[...] so naturally I assume that is still coronavirus so she encouraged me to take over the counter medication which I don’t do i’ve never done it I don’t do it I don’t believe in it I don’t have a Tylenol deficiency I don't have an aspirin deficiency i’m not ibuprofen deficient so I don't think I should take that.”

^a^CBD: cannabidiol.

^b^THC: tetrahydrocannabinol.

##### Taking Ownership of Treatment

The 3 related topics were “Alternate remedies,” “Dealing with uncertainties,” and “Not being listened to by physicians.” *Alternate remedies* dealt with long haulers sharing alternative medicine that they used and recommending alternative medicine to others. In *Dealing with uncertainties*, long haulers noted that they were dealing with symptoms for “an indefinite period of time*.*” On the basis of their experiences, they had an understanding that physicians were mystified by PCC and, thus, treatments were not certain or foolproof. This led to the last topic, which was “Not being listened to by physicians*.”* A recurrent topic thus far in the study, this dealt with patients not feeling listened to and supported by members of the health care system. One particularly popular account of this was shared by one woman in a video titled “I’ve had COVID-19 for a year. Here’s what I’ve learned.” She shared her experience as a woman and person of color who felt that she experienced particularly unfair treatment:

...there is so much research on patients reporting doctors not believing them or treating them with the same level of compassion.

Long haulers called for physicians to hold themselves accountable when confronting their own biases. If not, long haulers suggested that they were “violating the trust of their patients and trust is a key element to the patient physician relationship*.*”

##### Changes to Daily Life

Next, long haulers discussed the impact of the long haul on daily life. Associated topics included “Insomnia,” “How symptoms interrupt activities,” and “How symptoms present themselves.” Long haulers discussed how insomnia impacted their lives. They mentioned that their symptoms impeded their ability to exercise, eat foods they regularly ate, and even take showers. Finally, long haulers talked about how the symptoms initially presented themselves.

##### Choosing Homeopathy Over Pharmaceuticals

The 2 related topics were “Use of CBD and THC” for treatment and “Turning down over-the-counter medicine.” One long hauler looked to tetrahydrocannabinol gummies to cure insomnia in part because “I don’t like pharmaceuticals, I have never really liked them*.*” Other long haulers shared their apprehension about using pharmaceutical drugs and mentioned turning to more natural options instead.

### RQ 2 Results: How Do Users Respond to PCC Content?

#### Overview

To understand how users respond to PCC content, we separated comments for each category (news sources, medical sources, and long haulers) into 2 subcategories based on sentiment (negative and positive). We then used Biterm to generate topics for these subcategories. When looking at all sources combined, there was not a large discrepancy between the number of positive and negative comments. Overall, there were 1463 positive comments and 1382 negative comments.

However, when we began to look at the split of positive and negative comments by source, we could see that news sources received a greater share of negative comments. There were 687 negative comments and 391 positive comments. In contrast, medical sources received more positive than negative comments. There were 528 negative comments compared to 730 positive comments. Finally, long hauler videos only showed a 13-point difference between the number of positive and negative comments. There were 261 positive comments and 248 negative comments.

In addition to capturing the comments themselves, we captured metadata associated with the comments. This included comment replies, comment likes, and video description. Comment likes and replies indicate the level of engagement that other YouTube users had with the comment posted. Medical source video commenters saw an average of 16.02 (SD 45.09) likes per comment. The most liked comment received 602 likes. The most replied to comment received 474 replies. Conversely, news source video commenters saw an average of 36.46 (SD 168.34) likes per comment. The most liked comment received 2520 likes. The most replied to comment received 184 replies. Finally, long hauler video commenters saw an average of 54.55 (SD 246.72) likes per video. The most liked comment received 4127 likes. The most replied to comment received 223 replies (Table 3).

**Table 3:** Comment metadata.

Source	Number of comment likes, mean	Most likes on a comment, N	Number of comment replies, mean	Most replies on a comment, N
Medical source videos	16.02	602	3.15	474
News source videos	36.46	2520	4.23	184
Long hauler videos	54.55	4127	3.06	223

#### News Source Video Comments

#### Overview

Table 4 shows the resulting topics and themes from positive comments found under news source videos.

**Table 4:** Results of positive comments in news source videos.

Theme and topic label		Keywords	Sample comments
**Extending empathy**			
	Relating to others	“People,” “get,” “think,” “symptoms,” “many,” and “felt”	“omg, i can totally relate.”
	Well wishes	“Everyone,” “really,” “hope,” “take,” “care,” “able,” and “want”	“It would be terrible to lose your ability to taste or smell. Here’s to hoping they improve soon.”
	Gratitude	“Better,” “still,” “hope,” “people,” “feel,” “heart,” “help,” “something,” and “good”	“Your story was gut-wrenching, but still worth the share. Thank you. People need to hear this.”
**Expressing distrust through sarcasm**			
	Sarcasm	“See,” “think,” “often,” “say,” “real,” and “know”	“Okay so they survived a cold like most do. With a 99.8% survival rate, I’m sooo surprised.”
**Encouragement for better outcomes**			
	Prayers and scriptures	“Unto,” “shall,” “ye,” “people,” “peace,” “hath,” “forgive,” “love,” “reward,” “presence,” “pray,” “temple,” and “holy”	“Phillippians 4:7—And the peace of God, which surpasses all comprehension, will guard your hearts and your minds in Christ Jesus.”
	Potential solutions and sharing symptoms	“Ask,” “receive,” “keep,” and “know”	“Leronlimab is in clinical trials you guys. Don’t worry, help is on the way.”

#### Extending Empathy

*Extending empathy* comprised the topics “Relating to others,” “Well wishes,” and “Gratitude.” Comments in which people related to others involved people explicitly sharing that they related to the content shown or explaining how their symptoms were similar to those of the people interviewed in the news segments. For example, one commenter wrote the following:

You are not alone. I had COVID in April 2020 [...] I am currently in pulmonary rehab [...] I want others to know you are not alone. I’m praying for everyone. God Bless.

*Well wishes* was the second topic in this theme. In this topic, commenters sought to verbally empathize with those experiencing negative COVID-19–related symptoms:

Too bad for that young man, hopes he gets better!

Finally, in the *Gratitude* topic, commenters were also grateful that PCC content was being shared at all:

So glad she is sharing her struggles.

#### Expressing Distrust Through Sarcasm

Although the comments observed in this analysis were rated neutral or positive by VADER, some comments seemed to take on a sarcastic tone. For example, one commenter wrote the following:

The greatest nation in the world is your imagoNATION.

These sarcastic comments often appeared to exhibit political or skeptical undertones.

#### Encouragement for Better Outcomes

The topics within the *Encouragement for better outcomes* theme were “Prayers and scriptures” and “Potential solutions and sharing symptoms.” Many commenters left prayers and extensive Bible verses underneath videos as a form of encouragement for those battling PCC:

God heal these people from this virus. Give them strength.

Finally, *Potential solutions and sharing symptoms* was a topic that covered suggestions that commenters made to improve the symptoms of those dealing with PCC as well as sharing symptomatology in general:

Leronlimab is in clinical trials you guys. Don’t worry, help is on the way.

#### Negative News Source Comments

Table 5 shows the resulting topics and themes from negative comments found under news source videos.

**Table 5:** Results of negative comments in news source videos.

Theme and topic label		Keywords	Sample comments
**Reproduction of debunked and political theories**			
	Conspiracy theories	“Vaccine,” “face,” “different,” “affected,” “system,” “situation,” “avoid,” “dreadful,” “resist,” and “corona”	“This is all because of 5G poisoning.”
	Political influences	“Capability,” “dreadful,” “never,” “responsible,” “fight,” and “answer”	“They got their butts kicked by Kung flu.”
**Misinformation and disinformation**			
	Fear of impending doom	“Never,” “stress,” “know,” “affected,” “death,” “worry,” and “stop”	“Something’s coming, and we won’t be able to stop it.”
	Skepticism or rationalization	“Already,” “must,” “know,” “affected,” “response,” “another,” “nothing,” and “personal”	“Elderly people are susceptible to viruses. This is well known.”
**Issues with the health care system**			
	Not believed	“Medical,” “sick,” “normal,” “pain,” “feeling,” “never,” “anxiety,” “help,” “see,” “hope,” and “think”	“My primary care physician doesn’t believe me either [...].”
	Other illnesses	“Sick,” “heart,” “pain,” “long,” “time,” “blood,” “fatigue,” “brain,” “chronic,” and “help”	“I had this for decades with me/cfs. Imagine dealing with it for that long [...].”

#### Reproduction of Debunked and Political Theories

This theme comprised 2 topics: “Conspiracy theories” and “Political influences.” As an example of the *Conspiracy theories* topic, one commenter offered alternate causes of PCC symptoms, which were based on public disdain for mask wearing—“‘Long-haulers’ may actually be suffering from effects of prolonged mask-wearing […]”—instead of on veritable information. In contrast, *Political influences* covered suspected country or political involvement that contributed to the pandemic. When referring to individual damages incurred due to PCC, one commenter wrote the following:

...take the cost off the debt to china.

#### Distrust of Information Shared

This theme comprised 2 topics: “Fear of impending doom” and “Skepticism or rationalization.” *Fear of impending doom* comprised comments that pointed to a grim future for long haulers or the public:

...they’re just trying to kill all the long haulers when all you need is some ivermectin

...

Skepticism or rationalization comprised commenters who were not convinced that the information presented on PCC was veritable:

...they had flu colds bacterial lung infections pneumonia, many caused by face mask, no sunlight, fear and confinement

...

#### Issues With the Health Care System

This theme comprised 2 topics. “Not believed” covered comments condemning health care workers for dismissing the symptoms of their patients:

...typical doctor behavior: when in doubt, blame anxiety.

*Other illnesses* covered comments in which people drew similarities between PCC and other chronic illnesses:

This is so real...the Lyme community feels all your pain. And being denied by Dr’s that this is real. Its criminal to ignore this.

#### Medical Source Video Comments

##### Overview

Table 6 shows the resulting topics and themes from positive comments found under medical source videos.

**Table 6:** Results of positive comments in medical source videos.

Theme and topic label		Keywords	Sample comments
**Appreciation of helpful content**			
	Gratitude	“Help,” “medical,” “doctors,” “hope,” “thank,” “much,” “understand,” and “positive”	“This is the first thing that I have seen that explains anything besides the news trying to sensationalize and leave out important details.”
	Health literacy	“Need,” “information,” “understand,” “research,” “know,” “specific,” and “narrative”	“Your lectures are always easy to understand. Thank you Dr.”
**Hope and encouragement**			
	Prayers	“Hope,” “believe,” “feeling,” and “days”	“Jesus loves you [...].”
	Voice of reason	“Help,” “know,” “say,” “think,” “test,” and “research”	“You’ve always cared, been of a sound mind, and shared such insightful information. Thank you.”
	Bravery	“Research,” “doctor,” “video,” “help,” “know,” “people,” “feel good,” “positive,” and “believe”	“Even though this subject is controversial, you’re still brave enough to comment on it. Thank you.”
**Exchange of helpful information**			
	Seeking additional information	“Symptoms,” “help,” “information,” “video,” “wonder,” and “please”	“Has the Dr. released the additional information?”
	Seeking translated information	“Please” and “videos”	“Can you please translate to Arabic?”
	Sharing helpful information	“Think,” “information,” “help,” “vitamin,” “research,” “medical,” and “may”	“Yesterday, I saw an article that said we needed to be aware of [...].”

##### Appreciation of Helpful Content

This theme covered 2 topics: “Gratitude” and “Health literacy.” *Gratitude* covered general professions of thanks for the content shown. One commenter wrote the following:

Dr. Hansen, this is exactly the information I was hoping for! Thank you.

Health literacy in this case was covered in a positive light. Commenters thanked content makers for presenting information in a clear manner:

...as a lay person with zero medical background, I learn a lot.

##### Hope and Encouragement

This included 3 topics: “Prayers,” “Voice of reason,” and “Bravery.” *Prayers* included well wishes for those dealing with PCC or reading the comment section. This included requesting prayers as well:

Please pray for my mom...she is positive for covid 19.

The *Voice of reason* topic alluded to the idea that commenters deemed it important to find useful and truthful information:

Thank you for your commitment to keeping the world informed.

Finally, *Bravery* featured comments that alluded to the negativity that those sharing information about PCC and, more generally, COVID-19 face. One commenter noted the following:

...this subject is controversial and you’re still brave enough to comment on it.

##### Exchange of Helpful Information

This theme covered 3 topics: “Seeking additional information,” “Seeking translated information,” and “Sharing helpful information.” *Seeking additional information* featured those primarily asking questions such as the following: “What about cutaneous hyperestesia?” In *Seeking translated information*, many sought to understand content by having it translated into their native language. In *Sharing helpful information*, commenters tried to share what they deemed to be helpful to others:

Find a hyperbaric oxygen therapy chamber and a doctor checkup for compassionate use.

##### Negative Medical Source Comments

Table 7 shows the resulting topics and themes from negative comments found under medical source videos.

**Table 7:** Results of negative comments in medical source videos.

Theme and topic label		Keywords	Sample comment
**Negative impacts of the long haul**			
	Comorbidity	“Fatigue,” “disease,” “symptoms,” “pain,” “chronic,” “heart,” “brain,” “chest,” “feel,” “body,” and “diagnosed”	“How does this effect those with diabetes. I’m experiencing a range of symptoms.”
	Loss	“Family,” “end,” “life,” “months,” and “never”	“COVID-19 took my mom last year. I don’t know how I’ll move on.”
	Symptoms	“Symptoms,” “fatigue,” “disease,” “chest,” “heart,” “brain,” “taste,” “body,” “severe,” “hearing,” and “memory”	“I had a headache so bad that I had to seek treatment [...].”
**Requiring medical alternatives**			
	Criticism of physicians	“Doctor,” “bad,” “know,” “experience,” “need,” “study,” “poor,” “data,” and “must”	“These doctors have no idea what they’re doing. His advice makes no sense. I think we’ll be sick forever.”
	Debunked recommendations	“Study,” “poor,” “suffering,” “need,” “research,” “must,” and “know”	“How could you share so much but not talk about Ivermectin? You’re doing everyone an injustice.”
	Misinformation	“Vaccine,” “last,” “illness,” “death,” and “bad”	“Misinformation has gotten so bad that my own family won’t even believe me [...].”
**Lack of needs**			
	Lack of improvement	“Symptoms,” “feel,” “since,” “weeks,” “pain,” “back,” “effects,” “suffering,” “last,” and “vaccination”	“The vaccine didn’t improve my symptoms.”
	Lack of information	“Medical,” “would,” “think,” “cause,” “whether,” and “help”	“He mentions promising treatments but he never tells us what they are.”

##### Negative Impacts of the Long Haul

This theme comprised 3 topics: “Comorbidity,” “Loss,” and “Symptoms.” *Comorbidity* featured comments and questions that sought to relate PCC to other diseases:

Childhood obesity might be a factor

...

In *Loss*, some commenters spoke explicitly about those they lost to PCC or COVID-19. Finally, in *Symptoms*, commenters spoke candidly about the symptoms they faced:

I had a headache so bad that I had to seek treatment.

##### Requiring Medical Alternatives

In this theme, there were 3 topics: “Criticism of physicians,” “Debunked recommendations,” and “Misinformation.” In *Criticism of physicians*, commenters spoke about how they often felt dismissed by physicians when presenting their symptoms:

...if he went to visit my gp he would tell him he was stressed and it was in his head told me the same [...] it turned out to be lung scarring and a tumor.

In *Debunked recommendations*, commenters pushed for the use of medications that had already been proven to be not helpful and even toxic for human consumption. Ivermectin was notably one of these medications:

...should we be taking Ivermectin since our DNA now expresses spike protein forever?

Finally, *Misinformation* comments reverberated common antimask and antivaccine comments:

John do you have the list of ingredients of the vaccines? My daughter makes cupcakes and she has to list every ingredient by law...

##### Lack of Needs

This theme covered “Lack of improvement” and “Lack of information.” *Lack of improvement* largely related to symptoms not improving despite medical and home remedy attempts. *Lack of information* included criticism of content sources for not providing enough information regarding content (eg, treatment and research):

...he mentions promising treatments, but he never tells us what they are.

#### Long Hauler Video Comments

##### Overview

Table 8 shows the resulting topics and themes from positive comments found under long hauler videos.

**Table 8:** Results of positive comments in long hauler source videos.

Theme and topic label		Keywords	Sample comments
**Appreciation**			
	Bravery	“Sharing,” “thank,” “glad,” “feeling,” “believe,” “recovery,” “care,” “story,” “bless,” and “post”	“Your bravery hasn’t gone unnoticed. Thank you for all that you do.”
	Compliments	“Bless,” “share,” “thank,” “good,” “feel,” “positive,” “love,” and “believe”	“what a beautiful person inside and out.”
**Exchange of helpful information**			
	Seeking additional information	“Back,” “still,” “help,” “know,” “could,” “think,” “test,” “say,” “check,” “treatment,” “better,” “work,” “natural,” “support,” and “right”	“...eliminating carbs could potentially make things better. That’s what worked for me [...].”
	Sharing additional information	“Support,” “scheduling,” “doctor,” “right,” “treatment,” “better,” “work,” “great,” “different,” “try,” “may,” and “take”	“If you check my channel you’ll see why you should check your CRP. It could really help your lungs [...].”
**Community building**			
	Reaching out	“Need,” “people,” “help,” “sharing,” “video,” “time,” “want,” “research,” “appreciate,” and “experience”	“...is there any way that I can talk to you please or message you?”

##### Appreciation

The *Appreciation* theme comprised “Bravery” and general “Compliments.” Commenters lauded the content creator for being brave enough to share their experiences. This may allude to the idea that some who speak on their PCC experiences may face backlash. In addition, commenters gave content creators various accolades regarding their personalities and their decisions to share information:

...what a beautiful person, inside and out.

##### Exchange of Helpful Information

“Seeking additional information” and “Sharing additional information” were the 2 topics in this theme. Commenters often initiated or tried to engage in dialogue about topics such as potential treatments and tests for PCC symptoms:

...if you check my channel, you’ll see why you need to check your CRP.

##### Community Building

“Reaching out” was the topic in this theme. Commenters sought to connect with long haulers to continue conversations elsewhere.

##### Negative Long Hauler Source Comments

Table 9 shows the resulting topics and themes from negative comments found under long hauler videos.

**Table 9:** Results of negative comments in long hauler source videos.

Theme and topic label		Keywords	Sample comments
**Exchange of additional information**			
	Asking follow-up questions	“Get,” “think,” “covid,” “would,” “take,” “symptoms,” “different,” “know,” “since,” “less,” “help,” and “maybe”	“Are you still sick?”
	Sharing information via experience	“Symptoms,” “many,” “swollen,” “frustrating,” “lymph,” “body,” “covid,” “chest,” “get,” “pain,” “take,” and “help”	“You should check into your thyroid levels. I had issues with mine [...].”
	Seeking answers for symptoms	“Help,” “feel,” “usually,” “maybe,” “less,” and “symptoms”	“did anyone else experience long COVID anxiety?”
**Disillusionment with the health care system**			
	Disappointment with physicians	“Medical,” “pain,” “enough,” “nurse,” “support,” “right,” “hard,” “felt,” “know,” “fear,” “frustrating,” “people,” and “deal”	“Overachievers will never admit they don’t know something.”
	Unfair treatment	“People,” “frustrating,” “deal,” “problem,” “felt,” “hard,” and “know”	“...women and women of color are often treated this way. i’m not really surprised.”
**Requiring more visibility**			
	Gratitude	“Symptoms,” “sick,” “since,” “without,” “feeling,” “believe,” and “almost”	“My girlfriend has long COVID and she has so many of these issues.”
	Wanting more awareness	“Weeks,” “always,” “sick,” “bad,” “body,” “infection,” “low,” “feel,” “know,” and “symptoms”	“I barely see any information like this in the media. Why is that?!”

##### Exchange of Additional Information

“Asking follow-up questions,” “Sharing information via experience,” and “Seeking answers for symptoms” were the 3 topics in this theme. This theme was very similar to the theme that appeared in the positive long hauler comment analysis. There were slight differences between the examples in the 2 themes. The theme in this instance focused more on symptomatology in the case of the content creators or commenters:

...did anyone else experience long COVID anxiety?

##### Disillusionment With the Health Care System

The topics in this theme were “Disappointment with physicians” and “Unfair treatment.” In “Disappointment with physicians,” commenters mainly criticized the behavior of physicians in the context of PCC diagnosis or lack thereof. In addition, in “Unfair treatment,” commenters mentioned how specific groups may experience worse health care treatment than others:

...female patients and patients of color [...] there is so much research on patients reporting doctors not believing them or not treating them with the same level of compassion.

##### Requiring More Visibility

This theme comprised “Gratitude” and “Wanting more awareness.” Interestingly, although these comments were marked as negative, there were still a number of comments that expressed gratitude for the content creator sharing their message. This was often accompanied by sharing of their experiences as well. Relatedly, “Wanting more awareness” reflected the desire of commenters to see additional PCC content in the media, insinuating that there was not yet enough coverage.

## Discussion

### Principal Findings

#### Overview

Symptomatology was a prevalent theme across all sources. Video creators and commenters shared and empathized with each other regarding symptoms that occurred because of PCC. These symptoms included *prolonged fatigue*, *cognitive dysfunction*, *shortness of breath*, *cardiac issues*, and *lingering pulmonary symptoms*. This was consistent with the findings of several studies [4,14-16,33]. In medical source videos, medical professionals explained symptomatology and symptom etiology in both layperson and more scientific terms. In both news source and long hauler videos, personal experiences were shared, as well as how PCC symptoms had impacted their daily lives. Upon inspection of the comments, we found that symptoms were shared for a range of purposes. At times, it was purely to exchange knowledge and offer informational support. In addition, it was used as a means to connect with others to exchange emotional support [34].

#### Emotional and Informational Support

The positive themes identified in our findings can be operationalized as emotional and informational support. The emotional support category of themes comprised those in which commenters or video creators sought to empathize with others. This was through words of encouragement, prayers, sharing of similar experiences, and community building. Informational support themes covered themes in which users sought or shared information.

In both transcripts and comments, people discussed experiences of not being believed by physicians and having a perilous relationship with the health care system. This sentiment appeared to be common across the board; however, 3 groups stood out in particular: those with other chronic illnesses such as chronic fatigue syndrome and myalgic encephalomyelitis, women, and people of color. Those who had been battling chronic diseases for years before the emergence of PCC empathized with long haulers who felt that they were not being heard, as can be seen in Table 5. Complaints centered on being told that they were overexaggerating their symptoms or insinuations that patients were hypochondriacs (Table 5).

Women and people of color discussed how they felt dismissed by health care workers. There was a general sentiment of distrust. This notion has been backed by an NBC article, wherein one woman of color explained that she had been brushed off by physicians and labeled as aggressive [35]. This was despite the fact that she had lost 30 pounds and sight in her right eye as a result of PCC [35]. People of color have been disproportionately affected by PCC [35-37]. A total of 2 studies conducted by the National Institutes of Health [36,37] found that Hispanic and African American individuals had greater health problems and symptoms related to PCC but were less likely to be diagnosed. This corroborates anecdotal evidence from video comments (Table 9).

Though these topics occurred in comments with negative sentiment, there were positive repercussions: emotional and informational support. This general distrust of the health care system appears to have led to the adoption of homeopathic medicine, alternative medicine, and home remedies. In attempts to take their health into their own hands, users resorted to alternative treatments even if it put their freedom at risk. These comments were shared freely between video creators and commenters, exemplifying informational support. For example, one commenter noted that they smuggled marijuana into their state and felt that their insomnia had improved as a result of consuming it (Table 8). Others suggested changes in dietary habits (Table 8).

Another aspect of informational support dealt with health literacy. Health literacy was a theme that appeared most often in medical source–related videos. Health literacy has been defined by the Centers for Disease Control and Prevention as the degree to which individuals have the ability to understand and use information to make health-related decisions [38]. The content from medical sources exhibited 2 distinct tones. In the first case, information was delivered in layperson terms, which would likely be easier for the average person to understand. In the second case, scientists presented biological explanations of PCC in more jargon-filled language. There were mixed reactions. Commenters noted that, at times, they had issues understanding the content (Table 7). Issues with health literacy can impede one’s ability to properly advocate for themselves and understand what their options are. In other instances, commenters thanked the medical professionals for explaining PCC in a digestible manner, as can be seen in Table 6.

Symptom management was another topic that came up often in medical source and long hauler video transcripts and comments. In videos, medical professionals outlined steps that those with PCC could take to mitigate their symptoms (Table 10). In the comment section of medical source videos, commenters shared helpful information as well (Table 6).

**Table 10:** Medical source video transcript results.

Theme and topic label		Keywords	Sample transcripts
**Explanations in layman’s terms**			
	Symptomatology	“Symptoms,” “long,” “fatigue,” “common,” “brain,” “pain,” “loss,” “breath,” “chest,” “shortness,” “smell,” “body,” “fog,” “taste,” “breathing,” and “cough”	“Cognitive impairments things like word finding difficulty, short-term memory loss, difficulty with multitasking, poor concentration as well as anxiety and PTSD especially in patients who have been hospitalized.”
	Symptom etiology	“Syndrome,” “severe,” “illness,” “chronic,” and “different”	“[...] As I said earlier all of these symptoms, the headache, the sleep disturbance, the brain fog, they often tend to run together and sometimes it’s hard to say as to what is leading to what other symptom. It’s sort of like the chicken and the egg analogy. Is it because somebody has poor sleep, is that what leads to headaches because we do know what headaches can be triggered when the sleep is poor.”
	Symptom management	“Vitamin,” “time,” “day,” “sleep,” “work,” “different,” “need,” “help,” and “right”	“I think the first treatment for that insomnia is really sleep hygiene so that’s things like um turning off devices a half an hour before bed time, making sure you go to bed at the same time with a relaxing bedtime ritual, waking up at the same time every day, shutting devices off. [...]”
**Show housekeeping**			
	Introducing the show or guest and validating the guest’s credentials as a reliable source	“Dr,” “going,” “thank,” “time,” “want,” “talk,” “need,” “help,” “work,” “research,” “medical,” and “information”	“And he has organized several conferences given many lectures and has done live surgeries as demonstrations in several international conferences and forums [...] and nhs hospitals in UK [...].”
	Encouraging the audience to keep in touch	“Question,” “talk,” “help,” “data,” “group,” “better,” and “information”	“[...] There’s a conversation on X hashtag covered science um and uh all that remains then is for me to thank everyone that’s submitted questions. I hope I got through as many as I could.”
**Biological explanations**			
	Immunophenotyping	“ccr5,” “data,” “number,” “antigen,” “cd16,” “cd14,” “interleukin,” “dotted,” “chord,” “monocytic,” and “interstitial”	“[...] You know we can apply these variants in multiple applications, such as immunophenotyping cell sorting and also to study cell physiology [...].”
	Explaining the mechanics of immune responses	“Disease,” “percent,” “inflammation,” “severe,” “heart,” “illness,” “brain,” “course,” “study,” and “viral”	“[...] What’s really interesting is interleukin-2 and interferon-gamma are two cytokines that are intimately involved in antiviral immune responses and they are low in active because it’s an emerging infection our immune system presumably has not seen that virus before [...] so long covid actually has an immune response with high interferon-gamma that looks very much like a typical antiviral immune response.”

As implied, emotional support was operationalized as comments that extended empathy and compassion. This could often be found when there were accounts of personal experiences. Bible verses were shared as a means of offering hope. Commenters also thanked creators for sharing their story and offered prayers (Table 8). There was support from those living with other long-term illnesses, notably those with chronic fatigue syndrome or myalgic encephalomyelitis*.* Such discourse often led to community building in the comment section. This was particularly prevalent in the comment section of long hauler videos. To continue discussion, commenters asked follow-up questions regarding the progression of symptoms (Table 9). In addition, they sought other avenues to connect with and support each other (Table 8).

#### Skepticism, Misinformation, and Negatively Charged Comments on News and Medical Source Videos

We also observed a high frequency of negatively charged content, particularly in the comments for news and medical source videos. Skepticism regularly appeared in news-related content. Theories suggested by prominent politicians abounded, such as ivermectin as a cure for COVID-19. Many also criticized the credibility of the news sources and their supposed neutrality. News stations and reporters were, at times, labeled as people pushing liberal agendas and fear-mongering propaganda.

Misinformation and disinformation were major themes in both medical source and news source videos and comments. Some commenters felt that physicians were not sharing correct information or were misinterpreting the information that they had received (Table 7). This was despite the fact that, in many medical source videos, there was ample time spent expounding on the credentials of guest speakers, perhaps in an attempt to boost credibility before information was shared. In contrast, some commenters shared the opposite—they appreciated the scientific approach taken by physicians as opposed to news sensationalism (Table 6).

Other negatively charged comments dealt with the lack of needs: lack of information, lack of visibility, and lack of improvement. In general, commenters sought more information from health care professionals (Table 7). On a related note, commenters expressed wishes for more visibility regarding PCC. Commenters noted that their symptoms did not improve even once given the vaccine.

On the basis of our sentiment analysis, news source videos received by far the greatest proportion of negative comments. When assessing the topics and themes that came up in comments under news source videos, criticism and sharing of misinformation were dominant. Many of the ideas shared by commenters reflected those of politicians. In these views, blame for the spread of COVID-19 and COVID-19–based restrictions was shifted onto China and liberal politicians. Vaccine hesitancy and opposition expressed by commenters were reiterated by some politicians as well. Some commenters appeared to experience extreme fear with regard to the vaccine. They mentioned that those administering the vaccine and treating long haulers had motives to kill (Table 5). This seems to shed light on the idea that, although many previously debunked sentiments of politicians were being repeated, there was a genuine fear of vaccines, the health care system, and some members of the government. The sentiment analysis of videos from medical sources revealed that only a smaller portion (528/1258, 41.97%) of the comments were negative.

### Implications

The results of this study could help public health agencies, policy makers, organizations, and health researchers understand symptomatology and experiences related to PCC. The information includes a description of the diverse range of symptoms and informational and emotional needs of patients with PCC. This information can help public health professionals develop and implement effective interventions to manage PCC. Voices of Long Covid [39] is one campaign promoted by the US Department of Health and Human Services that emerged in November 2021 as a community for those with the syndrome. In addition to providing a forum for patients with PCC to share their experiences, the campaign offers resources for vaccinations and updates on developing research. The findings of this study demonstrate the potential of computational analysis of social media to provide insights and communication strategies regarding the public’s responses to future health crises. This can be used to provide additional perspective and information to such campaigns.

As referenced in the NBC News article [35], there are patients with PCC who have been met with resistance by some medical professionals. For example, one patient felt that she was dismissed after explaining her PCC symptoms. This dilemma has led to the creation of long hauler support groups on various social media platforms [35]. By mining YouTube, a rich source of our daily experiences, we began to uncover multifaceted challenges faced by long haulers. Our findings align with the experiences of patients who have lost work due to PCC and are unable to receive insurance coverage.

### Limitations and Future Work

There are some limitations to this work. Our study was conducted on YouTube transcripts. In many cases, transcripts for YouTube videos are automatically generated. This means that the captioning process is imperfect and, at times, incorrect words were recorded instead of the words that the speakers said.

In addition, we only reviewed top-level comments related to our videos, and our analyses on comments does not reflect the full scope of the discourse in the comment section. Thus, we may be missing important insights from responses to the comments. Future studies should extend this study to include reactions to comments as well. Another limitation is that we cannot assume that the comments presented underneath the videos in our study are representative of all viewers. Many viewers do not comment on videos [40]; thus, their opinions are not captured.

It is difficult to detect sarcasm and linguistic nuances using LDA and sentiment analysis. Despite this, sarcasm is often used in everyday speech. Because of this, the computational models may have interpreted some texts differently from how they were originally intended.

Future research could focus on the longitudinal experience of long haulers to examine how they are perceived and their overall experience over time. Long hauler sentiments toward the health care system and physicians could potentially have changed over time. In addition, as more information has surfaced and more COVID-19 infections have likely led to more PCC cases, there may have been a change in the level of skepticism and distrust when it comes to long hauler experience. Longitudinal studies would be able to explore this shift in their experience. Future research could explore the effectiveness of various public health strategies in mitigating the impact of PCC considering potential changes in public awareness and understanding fostered by increased media coverage, including YouTube.

Regarding recent PCC treatments, we started our research before drugs such as Paxlovid received full Food and Drug Administration approval on November 2023 [41]. We collected the videos on November 1, 2021, which included videos made in August 2020 after the spread of COVID-19 and until October 2021. A future study should investigate how the availability of PCC treatments changed the perceptions, management, and psychological impact of PCC.

It is important to acknowledge that the commenters and video creators in our YouTube study may be subject to selection bias and have excluded certain geographic and demographic perspectives. These perspectives hold some weight in how public sentiment should be perceived [42-45]. However, >95% of the internet population spanning 88 countries regularly interacts with YouTube [46]. This highlights the potential opportunity for broader exploration.

### Conclusions

In this study, we used topic modeling to investigate videos concerning PCC on YouTube. In addition, we assessed public responses to these videos by analyzing the comment section using sentiment analysis and topic modeling. We found that videos mostly focused on symptomatology, potential treatments, and sharing experiences. There was a range of response types, with news source videos receiving the highest proportion of negative comments and medical source videos receiving the lowest proportion of negative comments. Some were negative and often referenced conspiracy theories and distrust of the shared content. They also included negative experiences regarding PCC symptoms and treatment. Positive comments were those that exhibited community building, sharing of information, and offering of support. This information, which is based on social media analyses, can assist public health professionals in comprehending the responses to PCC, includes a description of the diverse range of symptoms and informational and emotional needs of patients with PCC, and can help public health professionals develop and implement effective interventions to manage PCC. The findings of this study demonstrate the potential of computational analysis of social media to provide insights and communication strategies regarding the public’s responses to future health crises.

## Abbreviations

LDA: latent Dirichlet allocation
PCC: post–COVID-19 condition
RQ: research question
VADER: Valence Aware Dictionary and Sentiment Reasoner

## Data Availability

The data sets generated during and analyzed during this study are available from the corresponding author on reasonable request.
